# Cytolytic Properties and Genome Analysis of Rigvir^®^ Oncolytic Virotherapy Virus and Other Echovirus 7 Isolates

**DOI:** 10.3390/v14030525

**Published:** 2022-03-04

**Authors:** Eero Hietanen, Marika K. A. Koivu, Petri Susi

**Affiliations:** 1 Institute of Biomedicine, University of Turku, 20520 Turku, Finland; eevahi@utu.fi (E.H.); mkahak@utu.fi (M.K.A.K.); 2 Turku Doctoral Programme of Molecular Medicine, University of Turku, 20520 Turku, Finland; 3 Turku Bioscience Centre, University of Turku, 20520 Turku, Finland

**Keywords:** Rigvir^®^, echovirus, virotherapy, cancer

## Abstract

Rigvir^®^ is a cell-adapted, oncolytic virotherapy enterovirus, which derives from an echovirus 7 (E7) isolate. While it is claimed that Rigvir^®^ causes cytolytic infection in several cancer cell lines, there is little molecular evidence for its oncolytic and oncotropic potential. Previously, we genome-sequenced Rigvir^®^ and five echovirus 7 isolates, and those sequences are further analyzed in this paper. A phylogenetic analysis of the full-length data suggested that Rigvir^®^ was most distant from the other E7 isolates used in this study, placing Rigvir^®^ in its own clade at the root of the phylogeny. Rigvir^®^ contained nine unique mutations in the viral capsid proteins VP1-VP4 across the whole data set, with a structural analysis showing six of the mutations concerning residues with surface exposure on the cytoplasmic side of the viral capsid. One of these mutations, E/Q/N162G, was located in the region that forms the contact interface between decay-accelerating factor (DAF) and E7. Rigvir^®^ and five other isolates were also subjected to cell infectivity assays performed on eight different cell lines. The used cell lines contained both cancer and non-cancer cell lines for observing Rigvir^®^’s claimed properties of being both oncolytic and oncotropic. Infectivity assays showed that Rigvir^®^ had no discernable difference in the viruses’ oncolytic effect when compared to the Wallace prototype or the four other E7 isolates. Rigvir^®^ was also seen infecting non-cancer cell lines, bringing its claimed effect of being oncotropic into question. Thus, we conclude that Rigvir^®^’s claim of being an effective treatment against multiple different cancers is not warranted under the evidence presented here. Bioinformatic analyses do not reveal a clear mechanism that could elucidate Rigvir^®^’s function at a molecular level, and cell infectivity tests do not show a discernable difference in either the oncolytic or oncotropic effect between Rigvir^®^ and other clinical E7 isolates used in the study.

## 1. Introduction

Since the discovery of viruses more than 120 years ago, they have attracted considerable interest as possible agents of tumor destruction. Early case reports showed the regression of cancers during naturally acquired virus infections; In most cases, viruses were arrested by the host immune system and failed to impact tumor growth, but sometimes, e.g., in immunosuppressed patients, tumors regressed [1,2]. Since the early days, reverse genetics and other technical advances allowed for the modification of numerous viruses, resulting in the concept of oncolytic virotherapy drugs. These virotherapy drugs aim to use native or modified viruses to mediate tumor regression through selective replication internally, and the lysis of tumor cells and/or induction of systemic antitumor immunity. As such, these virotherapy drugs are capable of eradicating tumors at distant, uninjected sites. Despite the technical advances, the mechanisms of action and efficacy of most viruses tested in oncolytic virotherapy have remained elusive, and consequently, there is only a single U.S. Food and Drug Administration (FDA) or European Medicines Agency (EMA) approved oncolytic virotherapy drug (genetically modified type I herpes simplex virus) available in global markets [3,4].

Interestingly, several human enteroviruses have shown promise in clinical trials against multiple cancer types [5,6]. One of the native picornavirus-based oncolytic virotherapy agents, CAVATAK^®^, which is cell-adapted coxsackievirus A21 (CVA21), has increased affinity to a decay-accelerating factor (DAF) cell surface receptor, as opposed to to native CVA2, and has the ability to infect several cancer types with cytolytic outcomes [7,8,9]. CAVATAK^®^ formulation has attracted the attention of BigPharma, and the startup that developed the virus was purchased by Merck Co. in 2018. Another human picornavirus, echovirus 7 (E7), has also been used as a backbone virus to develop an anti-cancer drug virus formulation named Rigvir^®^. E7 belongs to the Enterovirus B (EV-B) species (genus *Enterovirus*) within family *Picornaviridae*, which contains some of the most common viral pathogens of vertebrates [10,11]. It is common in epidemiological surveys and among the most common clinically diagnosed enteroviruses [12,13,14]. Similar to other human enteroviruses, E7 capsids are small, icosahedral and nonenveloped. They have 60 copies of four viral proteins—VP1, VP2, VP3, and VP4—that form an icosahedral shell with a diameter of 30 nm filled with a positive-sense, single-stranded RNA genome. The DAF receptor was identified as cellular receptor for the clathrin-mediated endocytosis and subsequent translocation of E7 to the site of replication within cell cytoplasm [15]. E7 multiplication results in the release of intact virus particles by cytolytic cell disruption in experimental cell lines [15,16].

Rigvir^®^ is a melanoma cell-adapted (genetically unmodified) formulation of echovirus 7 (E7) isolate. Rigvir^®^ claims to have both oncolytic and oncotropic properties, while being safe to use and free from adverse effects to the patient [17,18]. Although there have not been conventional clinical trials using Rigvir^®^ (https://www.clinicaltrials.gov/, accessed on 19 February 2022), the virus preparation was approved and registered in 2004 in Latvia for melanoma therapy, and shown to prolong the survival of not only melanoma stage IV M1c patients, but also small-cell lung cancer stage IIIA and histiocytic sarcoma stage IV patients in a limited number of case studies [19,20]. Additionally, it has been made available for an alternative treatment of melanoma and other cancers in other countries (https://hope4cancer.com/, accessed on 19 February 2022). Reportedly, Rigvir^®^’s approval as a virotherapy agent was withdrawn in Latvia in 2019 by Latvia’s State Agency of Medicines due to discrepancies with the laboratory testing of Rigvir^®^ samples and previously reported results. However, Rigvir^®^’s marketing website has no information about the withdrawal, and still lists the drug being approved in Uzbekistan, Georgia, and Armenia.

Even though Rigvir^®^ is claimed to be effective against many cancer cell types, including melanoma, small-cell lung cancer and sarcoma [19,20], cellular and molecular data about it are limited, and most background information regarding the virus is available only in Russian. In this report, we provide data for phylogenetic relationships and genome and structural comparisons between Rigvir^®^ and other E7 isolates, and compare cell infection profiles of Rigvir^®^ and five other E7 isolates, including the prototype Wallace sequence, from our clinical virus collection.

## 2. Materials and Methods

### 2.1. Viruses and Virus Culture

Viruses used in this study include echovirus 7 isolated from the original “Rigvir^®^” ampule (Sia Latima Ltd., Riga, Latvia), and viruses from our laboratory collection (E7 Wallace prototype from ATCC and four clinical E7 isolates originating from Finland (98-57213, 98-59065, 98-60628, and 07VI447)). All viruses were typed as “echovirus 7” according to the typing rules set by the International Committee on Taxonomy of Viruses (ICTV) Picornaviridae Study Group [11]. To determine rough median tissue culture infectious dose (TCID50) values and prepare stock viruses used in the further cell infectivity, viability and imaging assays, E7 isolates were inoculated onto Rhabdomyosarcoma (RD) cells, and cell lysate stock viruses were collected three days post-infection based on cytopathic effect (CPE).

### 2.2. Cell Lines Used to Test Infectivity and Viability

Human foreskin fibroblasts (HFF) were maintained in M199 medium supplemented with 5% fetal bovine serum (FBS) and 10 µg ml^−1^ gentamicin. Human bronchial epithelial cell line (16HBE14o) was maintained in Minimum Essential Media (MEM) with 10% FBS, 2 mM L-glutamine and gentamicin [21]. Human cervical cancer (HeLa Ohio) cell line was maintained in Basal Medium Eagle (BME) with 7% FBS and gentamicin. Human epithelial lung carcinoma (A549) cell lines were maintained in HAM’s F12 medium with 7% FBS and gentamicin. RD, human glioma (U373MG), human hepatocarcinoma (Huh7), human colorectal adenocarcinoma (SW480) and human breast cancer (MCF-7) cell lines were maintained in Dulbecco’s modified Eagle’s medium (DMEM) supplemented with 10% (FCS) and 10 µg mL^−1^ gentamicin.

### 2.3. Virus Infectivity and Viability Assays

Cells were seeded at 10,000 per well and grown on 96-well plates (PerkinElmer Health Sciences, Turku, Finland) to 60–80% confluency. Viruses were titrated at 1:10 intervals to reach optimal infectious dose. This was carried out to avoid cytotoxicity effects of cell lysate virus stock. Cell were visually inspected for signs of cytopathogenicity (CPE) for 3 days post-infection. Subjective terminology with (−; no cytolysis), (−/+; borderline result), (+; few cells lysed), (++; 50% of cells lysed) and (+++; full cytolysis of cells) were used to determine the extent of cytopathogenicity.

### 2.4. Immunofluorescence Microscopy

Cells were seeded at 10,000 per well and grown on 96-well plates (PerkinElmer Health Sciences, Turku, Finland) to 60% confluency. Viruses were inoculated onto cells, and unbound virus was removed after 1 hour (h) of incubation by washing three times with medium. Virus inoculum was defined as the volume that resulted in approximately 50% cell infectivity (TCID50) in RD cells based on the titration assay. Fresh medium was added, and the cells were incubated at 37 °C. The infection was allowed to proceed for 6 h, after which the cells were fixed with 4% formalin and permeabilized with 0.2% Triton X-100. Infected cells were stained with monoclonal pan-enterovirus 9D5 antibody (Millipore, Burlington, MA, USA) followed by combined staining with Alexa Fluor 488-labeled secondary anti-mouse antibody and staining of the nuclei with DAPI (25 µg ml^−1^). Images were acquired using a Zeiss Axiovert 200 M microscope equipped with A-Plan 10×/0.25 Ph1Var1 objective (Zeiss, Oberkochen, Germany). Brightness and contrast levels of the images were adjusted with Fiji (ImageJ) [22] and Adobe Photoshop image analysis programs (Adobe Systems Inc., San Jose, CA, USA). Likewise, quantitative image analysis of the immunofluorescence images was carried out in Fiji (ImageJ) [22] by first performing noise and background removal on the individual image channels. Segmentation of the nuclei was performed automatically, and the results were manually checked for consistency. After watershedding, the particle analysis function in Fiji was then used together with appropriate size constraints to calculate the number of individual nuclei. The number of infected cells was determined as largely similar by segmentation and particle analysis. However, the segmentation was performed more manually in cases where the fluorescence channel signal did not result in a good segmentation result otherwise. In cases where a large portion of the cells were infected and manual segmentation was infeasible, Cellpose [23] was used to automatically segment the infected cells and to create a mask of the area. The resulting image mask was then manually checked and adjusted if needed. Afterwards, the mask of the infected cells and the segmented nuclei image were combined in the Fiji image calculator through the “AND” function. The image calculator resulted in an image showing only the areas where the infected cell mask and the nuclei segmentation images overlapped, resulting in a new segmentation of cell nuclei belonging only to the infected cells. The nuclei were then calculated as described before using the Fiji particle analysis function.

### 2.5. Isolation of Viral RNA and Determination of Cycle Threshold Values

Viral RNA was extracted from 150 µL volume of virus-infected RD cell lysates using E.Z.N.A vRNA kit (Omega Bio-tek, Norcross, GA, USA) according to the manufacturer’s protocol, and stored frozen at −80 °C until use. The specimens were analyzed using a real-time RT-PCR [24]. RT-PCR amplification was performed with conserved (5′ and 3′) picornavirus primers from the 5′ noncoding region of the genome [25]. PCR reactions were performed in a RotorGene 6000 instrument (Qiagen, Hilden, Germany) in 25 µL reactions containing 5 µL of the RT reaction product, QuantiTect SYBR Green PCR mix (Qiagen, Hilden, Germany), and 600 nmol/L of virus primers. The PCR protocol used consisted of the following steps: 95 °C for 15 min, followed by 45 cycles at 95 °C for 15 s, 65–55 °C for 30 s (touchdown 1 °C/cycle for the first 10 cycles), and 72 °C for 40 s (melt 72–95 °C, 0.5 °C/s). Relative Ct values were determined from the results for each sample, and sample volumes were adjusted to the same levels before inoculation onto cells. Sample RNAs were extracted at 1 h and 72 h time points and Ct values were determined as shown above. Results are presented as multiples of a 10-fold increase in virus amount between the time points, using the estimated 3.3 difference in Ct values to correspond to 10 times difference in copy number (in-house determination for Rotor-gene using E7 RNA copy number controls) [26].

### 2.6. Genome Sequences and Sequence Analysis

Viral genome sequences and their primary characterization have been previously described (GenBank acc. no. MH043132-MH043137) [27]. Data are also shown for the 24 full length E7 isolates obtained from GenBank for this study, including the prototype, Wallace [28]. Sequence and phylogenetic analyses were carried out using Geneious Prime 2022.0.1 (https://www.geneious.com, accessed on 19 February 2022). Sequence alignments within Geneious Prime were conducted using the MUSCLE [29] and MAFFT [30] plugins for nucleotide and amino acid alignments, respectively, with the suggested settings. Phylogenies were built using Geneious Tree Builder with bootstrapping, using 500 replicates and a support threshold of 70%. Additionally, echovirus 9 prototype sequence X92886 (strain Barty) was set as an outgroup of the phylogeny.

### 2.7. Structural Analysis of E7–DAF Interface and Rigvir^®^ Mutations

Structural analysis of the DAF region was carried out using UCSF ChimeraX v1.2 [31] using the crystal structure of E7 capsid proteins VP1-VP4 published previously by Plevka et al. (PDB ID 2X5I) [32]. Amino acid mutations unique to Rigvir^®^, as discovered through previous sequence analysis, were mapped onto the crystal structure against residues that were previously described to form the DAF–E7 contact interface. Additionally, the surface exposure of the mutations was analyzed to determine their potential ability to affect receptor binding through direct surface exposure.

## 3. Results

### 3.1. Sequence Analyses and Genome Comparisons

**Figure 1:**
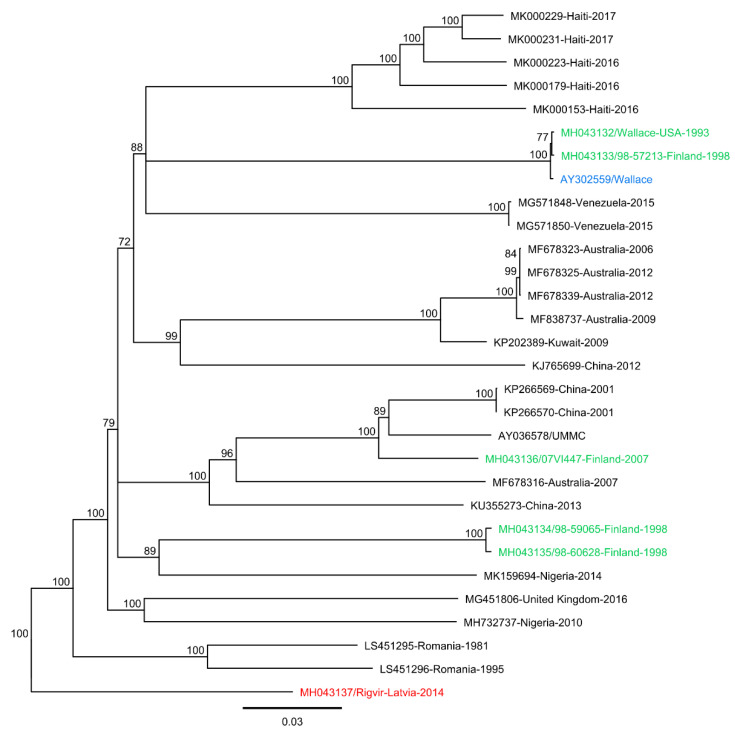
Neighbor-joining consensus tree of Rigvir^®^ and other full-length echovirus 7 (E7) sequences obtained from GenBank using 500 bootstrap replicates, with bootstrap values of ≥70% shown. Clinical isolates from this study are colored in green, Rigvir^®^ in red, and the prototype Wallace sequence in blue.

We have recently genome-sequenced Rigvir^®^ and other E7 isolates [27]. Preliminary genome analysis suggested both similarities and differences between the isolates. To further analyze the sequence data in respect to Rigvir^®^’s claimed properties, we performed sequence and phylogenetic analysis utilizing all full-length E7 sequence data available in GenBank. At present, there are 32 full-length E7 genome sequences in the GenBank (as of 6 December 2021), including our previously published 6 sequences [27]. All non-redundant (*n* = 30) full-length sequences were aligned using Geneious Prime software and the MUSCLE [29] and MAFFT [30] alignment plugins within the program. Phylogenetic analyses (Figure 1) were conducted using the Geneious Tree Builder program and revealed that the prototype Wallace (AY302559), originally isolated in the 1950s, was most closely related to isolate 98-57213. The isolate 07VI447 was found to be most closely related to the Chinese isolates KP266569 and KP266570 from 2001, as well as the isolate AY036578 (strain UMMC). Isolates 98-59065 and 98-60628 were found to be most closely related to a Nigerian isolate MK159694 from 2014. Rigvir^®^ was seen located in its own clade at the root of the phylogeny, most distant to any other full-length sequence used in the analysis. As Rigvir^®^ is a cell-adapted echovirus 7 strain, it is not surprising that sequence level and phylogenetic analyses indicate that it diverges from other echovirus strains. Mutations in Rigvir^®^ are likely caused by the original echovirus strain being subjected to multiple rounds of cell adaptation, with the aim of modifying the strain’s receptor affinity to target cancer cells (receptor-mediated adaptation). A similar approach was used, for example, for CAVATAK^®^ (coxsackievirus A21). Consequently, CAVATAK^®^ shows a higher affinity and enhances the rate of infectivity via the DAF receptor, which is widely overexpressed in many cancer cell types.

**Figure 2:**
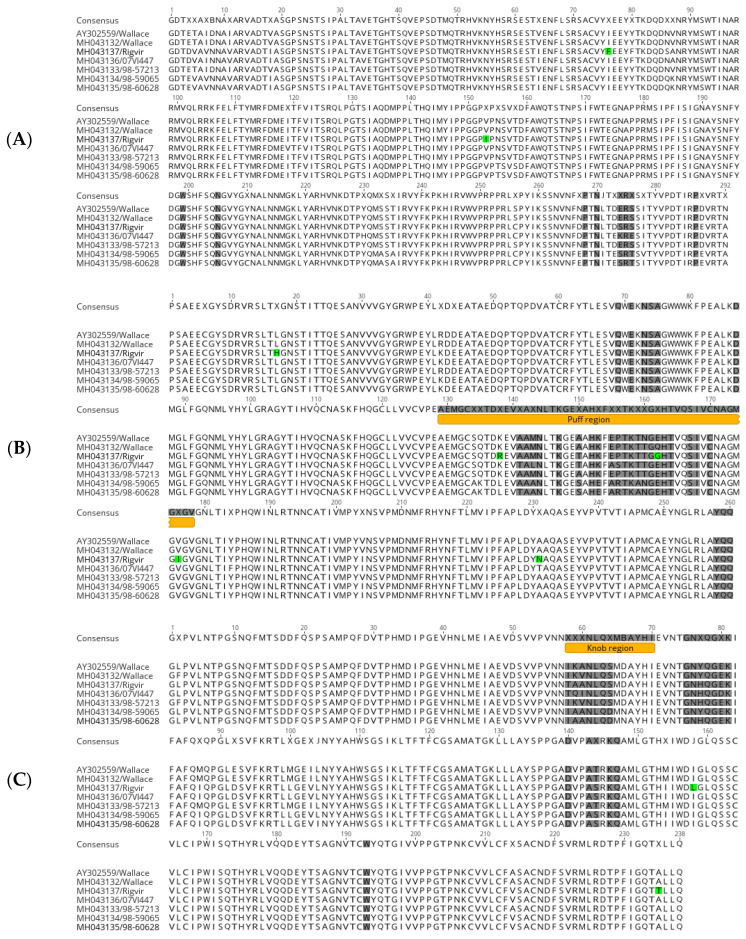
Protein sequence alignment of E7 capsid proteins: (**A**) VP1, (**B**) VP2, and (**C**) VP3. Positions shaded grey are residues that form direct contact with decay-accelerating factor (DAF). Unique mutations found in Rigvir^®^ are colored green, and the “puff” and “knob” structural motifs that contain the majority of DAF contact residues are colored orange.

While phylogenetic analysis is important for understanding the temporal and spatial differences between virus strains, further sequence comparisons at nucleotide and amino acid levels are important to reveal structural differences that are important in viral functions. Each echovirus 7 genome possessed a single large open reading frame (ORF) of 6585 nt, encoding a polyprotein of 2194 amino acids in length. When comparing the sequences to the prototype Wallace (AY302559), the whole genome nucleotide sequence identities ranged between 79.1% and 99.9%. Within the ORFs, the nucleotide identities, with respect to the Wallace prototype (AY302559), ranged from 78.9% to 99.8%, whereas the amino acid identities were highly conserved, ranging from 95.8% to 98.5%. Amino acid sequence pairwise identities between the viral capsid proteins VP1-VP4 ranged from 96% to 97.1%, with the lowest pairwise sequence identity occurring within the VP1 protein. The 5′ end of VP1 was especially variable, as well as a region roughly 20 nucleotides upstream of the 3′ end of VP1. Likewise, the pairwise amino acid identities for the non-structural proteins ranged from 92.6% to 98.7%. The lowest amino acid sequence identity occurred within the 2A protein, with amino acid variability seen throughout the 2A encoding region. A sequence analysis of unique mutations found in Rigvir^®^’s capsid proteins, VP1-VP4, resulted in a total of nine mutations (Table 1). Two mutations were found in VP1: I/L76F and V153I; five mutations in VP2: L17H, K/L/N138R, E/Q/N162G, V176I, and T/A231N; and two mutations in VP3: I158L and A235T. Positions of these mutations are reported with respect to the location in the E7 crystal structure (PDB ID 2X5I). Out of the previous mutations, one mutation, E/Q/N162G, was located in the residues previously described as forming the DAF–E7 contact interface [32]. Overall, the sequence analysis of the residues included in the E7 DAF contact region revealed that the region holds a considerable sequence variability, indicating a degree of flexibility in the DAF contact residues, which may be important in virus adaptation to a target cell line (Figure 2).

### 3.2. Structural Analysis of DAF Binding Site

**Figure 3:**
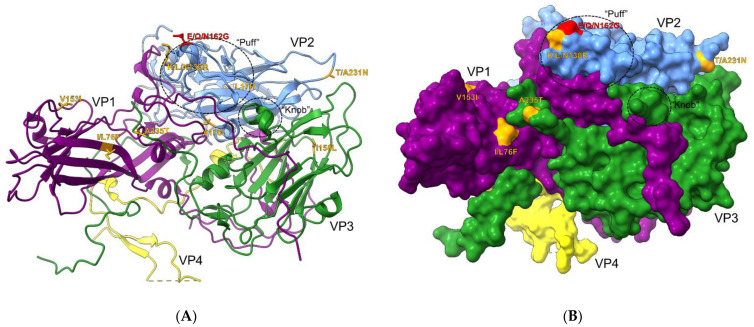
Echovirus 7 capsid protein structure (PDB ID 2X5I) depicting unique amino acid mutations. Circled “puff” and “knob” regions are known to hold majority of DAF contact residues. (**A**) Ribbon view of the asymmetric subunit with unique mutations found in Rigvir^®^ in orange. A mutation in red indicates a change directly in the DAF contact-forming residues. (**B**) Surface projection of the asymmetric subunit. Visible mutations in orange and red exhibit sufficient surface exposure.

The previous sequence and phylogenetic analyses were suggestive of some potential differences between Rigvir^®^ and other E7 isolates, which may further indicate differences in their ability to bind and infect cancer cell lines. To further examine the mutations present in Rigvir^®^’s capsid proteins, the discovered mutations were mapped onto the E7 crystal structure (PDB ID 2X5I) (Figure 3). Six mutations were found to have surface exposure on the cytoplasmic side (Figure 3B, Table 1). Additionally, two of these mutations, E/Q/N162G and K/L/N138R, were found to be in the previously described “puff” region of the VP2 capsid protein (Figure 3B). The “puff” and “knob” regions were previously described by Plevka et al. as containing the majority of the DAF contact interfaces with E7 [32]. Out of the two mutations, E/Q/N162G is also directly part of the residues described to form a contact with DAF. Additionally, these mutations can be structurally positioned in very close proximity to each other (Figure 3A). These results indicated that Rigvir^®^’s claimed properties could be due to subtle differences in receptor binding as a consequence of unique mutations located on the viral capsid surface.

### 3.3. Cell Infectivity and Viability Assays and Immunofluorescence Microscopy

An analysis of the E7/DAF contact interface and unique amino acid mutations found in Rigvir^®^’s sequence suggested that its claimed oncolytic and oncotropic properties could be explained by unique mutations found on the capsid surface. This may affect virus binding to the cell surface, and thereafter its ability to infect cells to cause cytolysis. To obtain a more conclusive view of how these mutations could affect infection in vitro, cell infection assays were performed using different native and cancer cell lines to follow cytopathogenicity. Viruses were first titrated on 96-well plates in RD cells (assuming similar infectivities) to determine individual TCID50 values between the isolates, and these values were then used at comparable levels to determine infectivities and cell viabilities in each cell line. Cell infection was determined by visual inspection 3 days post-infection and by immunofluorescence, and virus multiplication was verified by RT-qPCR. While most of the cells were infected based on the formation of cytopathic effect (CPE), there were no signs of infection in breast cancer cell line, MCF-7 (Table 2). Interestingly, two native cells lines, HFF and 16HBE14o, were also infected with cytolytic outcome, which undermines the claim of Rigvir^®^’s specificity to cancer cells.

**Figure 4:**
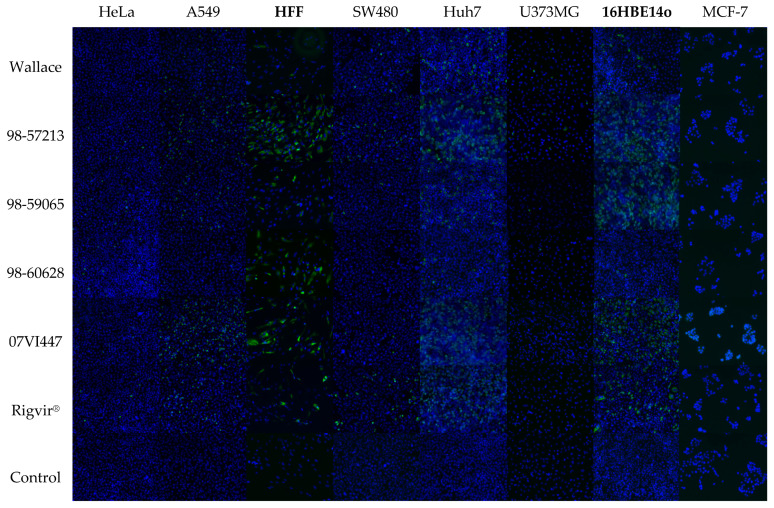
Immunofluorescence results of cell infection using TCID50 across six cancer and two native cell lines (bolded) with virus isolates used in this study. Rigvir^®^ is seen infecting both native and cancer cell lines, with an overall infection profile reminiscent of what is seen across clinical E7 isolates. MCF-7 cells are seen as non-infectious across all isolates in this study.

While it is possible that E7 infection in MCF-7 occurs without cytolysis, we also used immunofluorescence microscopy (IFA) to visualize virus entry and multiplication in the cell interior. This was also to confirm the CPE results because some cell lines, such as A549 and SW480, divide in a manner that resembles CPE (Figure 4). IFA results were consistent with the CPE results in that all viruses with clear CPE were also visualized in infected cells using IFA. Differences were observed in infectivity between cell lines, which may be explained by differential receptor expressions or the ability of the virus to replicate within the cells (Figure 4). Yet, there were also differences between the isolates in their relative infectivities, as is seen from the quantitative image analysis results of the immunofluorescence images as seen in Table 3. Huh7 was the only cell line in which a slightly pronounced infectivity of Rigvir^®^ was detected over the other E7 isolates based on the IFA results with 48.9% of cells infected. However, isolates 98-57213 and 07VI447 also both reached very high infectivities compared to other isolates: 36.6% and 40.6%, respectively. Breast cancer cells (MCF-7) were negative in IFA (Figure 4), and RT-qPCR (data not shown) suggesting that they are not susceptible to Rigvir^®^ or other E7 isolates. In the remaining cases, Rigvir^®^ is seen infecting the cell lines in question without major deviations from the trend set by the clinical E7 isolates; Rigvir^®^ is seen infecting cancer cell lines together with the clinical isolates, and not with a seemingly better efficiency. At the same time, Rigvir^®^ is also infecting non-cancer cell lines together with the other isolates. In summary, these data suggest that there is no clear difference between Rigvir^®^ and other echovirus 7 isolates in their ability to infect and lyse the target cells, both in terms of native and cancer cell lines.

### 3.4. Determination of Relative Infectivities by RT-qPCR

**Figure 5:**
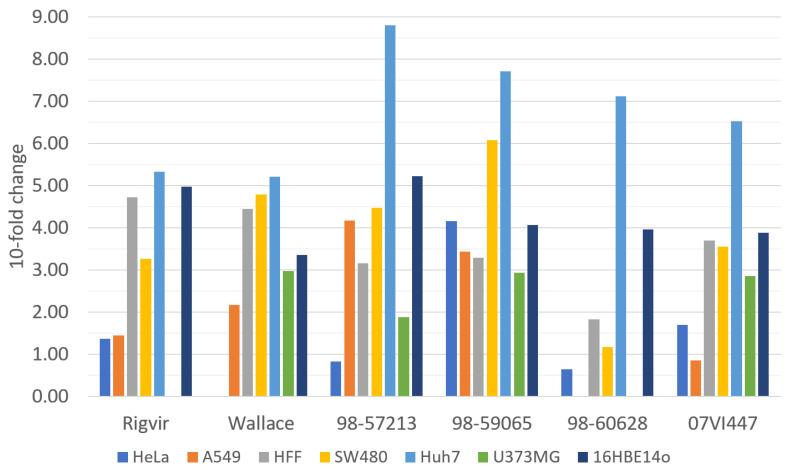
Number of 10-fold changes observed in virus amounts based on RT-qPCR Ct values of Rigvir^®^ and other echovirus 7 isolates between 1 h and 72 h time points.

Since Rigvir^®^ is cell-adapted variant of echovirus 7, it is possible that its cytolytic properties are more pronounced than those of the other E7s, and thus it has an increased its capacity to infect and lyse target cancer cells. While cell infectivity assays indicated which cell lines were susceptible to echoviruses, they did not reveal the relative differences in virus multiplication. To measure virus multiplication, the RT-qPCR method was used. Ct values were measured with a single replicate for each virus sample isolated from RD cells, and were used to adjust the sample volumes to equal levels prior to cell infection. Samples were collected from 1 h and 72 h time points post-infection, and the relative change in Ct values is reported as a 10-fold increase in the observed virus amounts (Figure 5). The results based on CPE were in line with the titration results; we did not detect any clear differences in the replication rate between the viruses.

## 4. Discussion

In this study, we monitored cell infectivities and performed further sequence and structural analyses to compare the properties of the Rigvir^®^ oncolytic virotherapy virus and other E7 isolates. In principle, there are two viral formulations for oncolytic virotherapy; cell-adapted viruses and genetically modified viruses. Cell-adapted viruses are usually subjected to several rounds of infection in target cell types to randomly increase binding specificity and increase replication rate, while gene-modified viruses are specifically modified to modulate cell specificity, replication rate and/or immunogenicity. While there were claims for clinical efficacy of Rigvir^®^, there are no data regarding the mode of action to warrant its use in oncolytic virotherapy. So far, the published results advocating for Rigvir^®^ focus on its cytolytic effects against different cancer cells. However, as echoviruses possess an innate ability to lyse cells through infection, the lack of comparative studies against native cell lines, as well as the inclusion of additional clinical E7 isolates, is a major flaw in the analysis of a claimed oncolytic and oncotropic virotherapy drug such as Rigvir^®^. Additionally, Rigvir^®^ is worryingly being offered as a treatment in “alternative cancer therapy” in some cancer institutes (e.g., Hope4Cancer Institute), or to treat terminal cancer patients in the EU.

Previously, it was demonstrated that Rigvir^®^ is capable of reducing the viability of human melanoma, rhabdomyosarcoma, gastric adenocarcinoma, lung carcinoma, and pancreas adenocarcinoma cells [33]. Cytolytic properties were proposed as mechanism of action for their clinical benefit. However, the type of analyses carried out in this study show that there is no evidence that Rigvir^®^ is different from other echovirus 7 isolates, including the virus isolates that circulate in the population.

It was shown that both non-structural and structural (protein-encoding) regions affect the infectivity of enteroviruses. For coxsackievirus B2 and EV-A71, pathogenicity determinants were shown to reside in VP1 capsid protein-encoding regions, respectively [34,35,36,37]. While serving as a cell surface entry receptor for several picornaviruses, DAF receptor is also broadly expressed in malignant tumors and plays a major role in promoting tumorigenesis [38]. Consequently, some picornaviruses were developed to make oncolytic virotherapy agents based on their ability to target and bind to DAF receptors. While many picornaviruses possess a distinctive capsid surface depression around the 5-fold axes of symmetry, called the “canyon” [39], as the site for receptor binding, some of the enteroviruses also use sites outside the canyon. The decay-accelerating factor (DAF) has been identified as a cellular receptor for the clathrin-mediated endocytosis of E7 [15]. The DAF binding site on E7 is near a 2-fold icosahedral symmetry [32]. Previously, the E7-DAF contact interface was mapped to 59 residues by Plevka et al., and majority of the residues were located in two regions, “puff” and “knob”, within the VP2 and VP3 proteins, respectively [32]. These regions of the capsid surface form the largest protrusions on the cytoplasmic side. Previous studies comparing DAF binding between E7 and E12 showed that there are four residues within VP2 that are both conserved between the virus species types, and exhibit significant enough surface exposure to be considered significant in DAF binding. These residues are Thr157, Gly161, His163, and Thr164.

Bioinformatic sequence analyses of the capsid proteins VP1-VP3 with Rigvir^®^ and other full-length E7 isolates show Rigvir^®^ as divergent from the rest of the isolates, with some unique mutations found in Rigvir^®^’s sequence. However, on an overall sequence level, individual, sequence-specific mutations are not exclusive to Rigvir^®^. A further phylogenetic analysis carried out with all of the available full-length E7 sequences found Rigvir^®^ to be the most distant compared to the rest of the isolates. Rigvir^®^’s phylogenetic divergence is expected due to the fact that Rigvir^®^ is a cell-adapted echovirus 7 strain that has undergone multiple rounds of cell-adaptation, with the original intention of increasing its affinity to melanoma cells. Additionally, regarding the DAF receptor binding interface with E7, sequence analyses show that the 59 residues belonging to the DAF contact interface are seen containing a considerable degree of amino acid variability. This would indicate a level of flexibility in the region, and thus individual mutations are difficult to claim as being responsible for such a drastic change in virus behavior, as is claimed for Rigvir^®^. Furthermore, none of the previously mentioned four residues that are considered to be significant in DAF binding were mutated in Rigvir^®^, although mutations were seen to occur in the residues adjacent to them. However, variability in the adjacent residues was not exclusive to Rigvir^®^ and was also seen in clinical E7 isolates.

Virus infectivity is primarily determined by two factors: receptor tropism and ability to replicate within cells. In this work, we aimed to compare relative virus infectivities in target cells using equal virus inocula. Since we do not have feasible methods to measure the number of virus particles in purified virus preparations, we used RT-qPCR to determine viral RNA copy numbers in samples collected from RD cells. Sample volumes were adjusted to equal levels based on copy numbers and prior to inoculation onto target cells. We collected samples at the 1 h and 72 h time points and calculated virus multiplication for each sample. As a result, we did not detect significant differences in relative infectivities between the samples across all cell lines (Figure 5). The viruses were also visualized using pan-enterovirus antibodies and cytolytic responses and were recorded using the naked eye based on cell cytopathogenicity, which is a general characteristic of many enteroviruses, including echovirus types [40]. We did not find any significant differences between the isolates in their abilities to infect, replicate and cause cytolysis in the target cell lines. Similar to the previous data published by other authors [33], our data show that E7 isolates infect both cancer and native human cell lines with cytolytic responses.

The action mechanism of Rigvir^®^ is currently unknown. Intriguingly, there are only a few clinical studies related to efficacy of Rigvir^®^ as an oncolytic virotherapy agent, and in those studies Rigvir^®^ was administered by muscular injections, that is, the effect is claimed to occur systemically. In addition, the virus is administered consecutively and in monthly intervals, which raises the question of immunogenicity and host response. Enteroviruses, including echoviruses, induce adaptive immune responses; therefore, it is difficult to conceive the need for consecutive injection during the treatment, since it is likely that the adaptive immune response generated after the first dose will halt the secondary infections. Further clinical experiments for demonstrating efficacy would benefit from virological and immunological analyses. In the light of the findings, the in vitro cell infectivity properties do not explain the anti-cancer activities of Rigvir^®^. Furthermore, the data regarding sequence variation and receptor use are inconclusive and insufficient for explaining the clinical benefit of Rigvir^®^ and calls for further studies to warrant the use of Rigvir^®^ or other E7 isolates in oncolytic virotherapy.

## Figures and Tables

**Table 1:** Unique amino acid mutations present in Rigvir^®^ capsid proteins. Sequence positions indicated as in echovirus 7 crystal structure PDB ID 2X5I.

Capsid Protein	Echovirus 7 Residue	Structure Position	Rigvir^®^ Residue	Surface Exposure
1	I/L	76	F	Yes
1	V	153	I	Yes
2	L	17	H	No
2	K/N/L	138	R	Yes
2	E/Q/N	162	G	Yes
2	V	176	I	No
2	T/A	231	N	Yes
3	I	158	L	No
3	A	235	T	Yes

**Table 2:** Cytopathogenicity (CPE) results 3 days post-infection using TCID50. Results are marked as CPE using terminology with (−; no cytolysis), (−/+, borderline result), (+; few cells lysed), (++; 50% of cells lysed) and (+++; full cytolysis of cells).

Isolate	GenBank Acc. No.	Cell Line							
		HeLa	A549	HFF	SW480	Huh7	U373MG	16HBE14o	MCF-7
Wallace	MH043132	−/+	+++	+++	++	+++	++	++	−
Rigvir^®^	MH043137	+	++	+++	+++	+++	−/+	+++	−
98-57213	MH043133	+	++	++	++	++	++	+++	−
98-59065	MH043134	+	+	++	+	+++	+	+++	−
98-60628	MH043135	+	+	+	−/+	++	−/+	+	−
07VI447	MH043136	+	+++	++	+	+	−/+	+++	−

**Table 3:** Infection efficiency based on quantitative image analysis of the immunofluorescence images. Infection efficiency is represented as a percentage of total number of cells in each image infected by the respective virus isolate.

Isolate	Cell Line							
	HeLa	A549	HFF	SW480	Huh7	U373MG	16HBE14o	MCF-7
Wallace	0.9	4.1	15.5	4.0	11.1	1.6	2.9	0.0
98-57213	2.9	19.4	83.5	7.3	36.6	5.2	39.2	0.0
98-59065	3.6	7.8	16.0	1.6	7.8	0.4	43.9	0.0
98-60628	2.7	4.6	43.7	0.5	2.4	0.4	0.3	0.0
07VI447	1.6	42.6	28.4	1.4	40.6	0.2	44.1	0.0
Rigvir^®^	1.1	9.3	31.4	7.1	48.9	0.4	11.7	0.0
Control	0.0	0.0	0.0	0.0	0.0	0.0	0.0	0.0

## Data Availability

Not applicable.
